# Peripheral blood eosinophilia in adult asthmatic patients and its association with the severity of asthma

**DOI:** 10.1186/s12890-023-02383-x

**Published:** 2023-03-22

**Authors:** Yenealem Solomon, Birhanemaskal Malkamu, Ayenew Berhan, Tahir Eyayu, Andargachew Almaw, Biruk Legese, Berhanu Woldu

**Affiliations:** 1 Department of Medical Laboratory Science, College of Health Sciences, Debre Tabor University, P.O. Box: 272, Debre Tabor, Ethiopia; 2 Department of Hematology and Immunohematology, School of Biomedical and Laboratory Sciences, University of Gondar, Gondar, Ethiopia

**Keywords:** Asthma, Eosinophilia, Severe asthma, Type2-low asthma

## Abstract

**Background:**

Asthma is a diverse disease with various etiologic bases. Severe asthma can be associated with increased mortality, hospitalization, and decreased quality of life for asthma patients. High blood eosinophil counts were associated with severe asthma, but recent studies have failed to confirm this as a marker of severe asthma among adult asthma patients. As a result, the purpose of this study was to determine the association between the severity of asthma and high blood eosinophil count.

**Methodology:**

A simple random sampling technique was used to select 291 asthmatic patients for an institution-based cross-sectional study. Socio-demographic, behavioral, and clinical characteristics were collected by using a pre-tested structured questionnaire. Four milliliters of venous blood were collected from asthmatic patients for complete blood count and peripheral morphology assessment. The eosinophil count was analyzed by the Unicel DxH 800 (Beckman Coulter, Ireland) analyzer. A statistical package for social science version 20 (SPSS) software was used to analyze the data. The non-parametric (Mann-Whitney U) test was used to compare the eosinophil count with different background variables. A binary logistic regression analysis was used to assess the factors associated with eosinophilia. A p-value less than 0.05 in multivariable logistic regression analysis was considered statistically significant.

**Result:**

In this study, the overall magnitude of eosinophilia was 19.6% (95% CI = 14.8–24.1). Being admitted to the emergency department (AOR = 0.25; 95% CI: 0.09–0.69, p = 0.007) and being female (AOR = 0.49; 95% CI: 0.26–0.9, p = 0.025) were shown to have a statistically significant association with eosinophilia. Moreover, the absolute eosinophil count was significantly higher among asthmatic patients infected with intestinal parasitic infection (p < 0.045).

**Conclusion:**

Being female and admission to the emergency department were negatively associated with eosinophilia. Lack of eosinophilia can be related to the low-T2 asthma phenotype. The absolute eosinophil counts were higher among intestinal parasite-infected patients. Therefore, different biomarkers will be considered for the proper diagnosis and management of adult asthma patients.

## Introduction

Asthma is a diverse disease characterized by chronic airway inflammation [1]. It is defined by a history of respiratory symptoms such as wheezing, chest tightness, shortness of breath, and cough that can fluctuate over time and in intensity along with variable expiratory airflow limitation [1, 2]. Asthma is a common disease of the airways that affects 339 million people worldwide [3]. Asthma prevalence is still growing in various countries [4]. In adults, the frequency of asthma ranges from 4 to 10% [4–6].

Asthma is a heterogenous disease with various etiologic bases [1, 7]. Asthma endotypes can be largely classified as type 2 (T2) high or T2-low [7]. T2-high asthma is distinguished by fractional exhaled nitric oxide (FeNO) > 25 ppb, blood and airway eosinophilia (peripheral blood eosinophil levels > 300 or > 150 cells/µL, and sputum eosinophils > 2%), increased severity, and therapeutic responsiveness to Glucocorticoids and T2 inflammatory inhibitors [8, 9]. During exposure to allergens, T2 inflammation coexists with eosinophilic inflammation mediated by cytokines such as interleukin (IL) -4, IL-5, and IL-13 [10, 11]. T2-low asthma is identified by increased neutrophils and a lack of airway and systemic eosinophilia [8, 10].

The European Respiratory Society (ERS)/American Thoracic Society (ATS) task force defines severe asthma as “asthma that needs treatment with high-dose inhaled corticosteroids (ICS) plus additional controller and/or systemic corticosteroids to keep it from being “uncontrolled” or it remains “uncontrolled” despite maximally optimized Global Initiative for Asthma (GINA) step 4 or 5 therapy and treatment of contributing factors, or it worsens when optimized therapy is reduced”. It is estimated that about 3 to 10% of asthma patients have severe asthma [1, 11].

Severe asthma is associated with higher mortality, hospitalization, and a decreased quality of life. Also, it results in physical, mental, emotional, social, and occupational costs for individuals as well as a financial impact on health care systems [12, 13].

Eosinophils are responsible for inflammatory effects when triggered by allergens. Persistent eosinophilic airway inflammation and airway remodeling lead to persistent airflow obstruction [14]. A high blood eosinophil count causes immune-modulatory responses such as airway inflammation, airway hyperresponsiveness, damage to the epithelial lining, and increased mucus secretion [15]. Nearly half of asthmatic patients have eosinophilic inflammation. Studies have shown that eosinophilia can be linked with increased disease severity, exacerbation frequency, and symptom burden, as well as impaired lung function [16–18].

Eosinophils are important predictors of disease severity and progression [19]. As a result, eosinophils play a critical role in asthma diagnosis. Moreover, eosinophil counts have emerged as a promising and easily measurable marker in eosinophilic airway inflammation [20, 21]. Although allergic sensitization has been identified as a risk factor for asthma [22], non-allergic asthma is more common in adults. The prevalence of allergic asthma is higher during early childhood and gradually declines with advanced age. Especially after the age of 40 years, the new cases are non-allergic asthma [23, 24].

The ERS/ATS task force considered sputum and blood eosinophilia as markers for severe asthma [11]. However, GINA stated that the blood eosinophil count is not employed as a diagnostic biomarker for asthma. Instead, it can be utilized as a prognostic biomarker, predicting therapeutic responsiveness and a diagnostic biomarker for defining asthma phenotypes in patients with type 2 inflammation [25]. Several cross-sectional and longitudinal studies have identified blood eosinophil counts as a risk factor for asthma exacerbation [26–28]. Conversely, recent follow-up studies found that eosinophil counts are not linked to asthma exacerbations [29, 30].

Many studies have revealed that eosinophil levels rise in cases of severe asthma. However, asthma is a diverse disease with different etiologies and phenotypes. Therefore, the main aim of this study was to determine the magnitude of the elevated peripheral eosinophil count and its association with the severity of asthma among adult asthmatic patients.

## Methods and materials

### Study design, area, and period

An institutional cross-sectional study was conducted from June to August 2021 in Northwest Ethiopia, at the University of Gondar Comprehensive Specialized Hospital (UGCSH) and Tibebe-Ghion Specialized Hospital (TGSH). UGCSH is located in Northwest Ethiopia, Amhara Regional State, in Gondar Town. The hospital can provide service for over 7 million people in the catchment area. There are over 400 regular follow-ups of asthmatic patients in the chronic outpatient department of the hospital. On the other hand, TGSH is located in Northwest Ethiopia, in Bahirdar City. The hospital provides health services for eight nearby zones in the Amhara regional state.

### Study participants

This study included all adult asthmatic patients (≥ 18 years old) attending UGCSH and TGSH during the study period. On the other hand, patients with known chronic diseases such as HIV/AIDS, hematological malignancies, chronic kidney disease, and tuberculosis were excluded from this study by reviewing their medical records.

### Sample size determination and sampling technique

#### Sample size determination

The sample size was determined by using the single population proportion formula by considering a proportion of 50%, 5% margin error, powe of 80%, and a 95% confidence interval. By using a formula, $n=\frac{{\left({Z}_{\frac{\alpha }{2}}\right)}^{2}p\text{q}}{{\text{d}}^{2}}$ = (1.96)^2^ (0.5 × 0.5)/(0.05)^2^ = 384.

Since the total number of asthmatic patients in two hospitals per year was less than 10,000 (N < 10,000). Thus, a reduction formula was used. So, $\text{n}=\frac{\text{n}\text{o}}{1+\frac{\text{n}0}{\text{N}} }$ = $\frac{384}{1+\frac{384}{1200} }$= 290.9 = Approx, n = 291

**Fig. 1:**
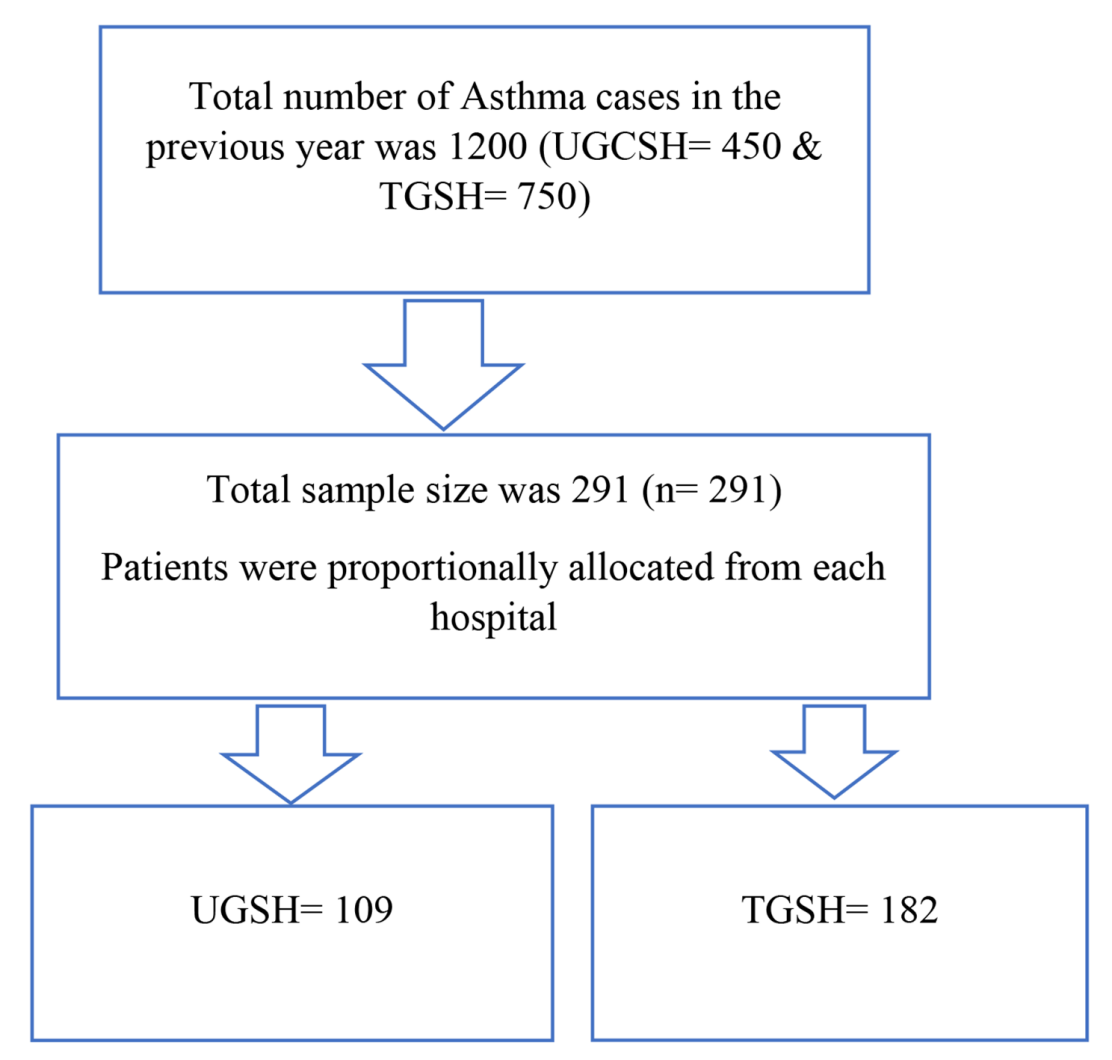
Sampling procedure

The study participants were proportionally allocated from each hospital (Fig. 1). The lottery-based simple random sampling technique was used to select the study participants.

#### Operational definitions

- **Asthma**: any person who has been diagnosed with asthma by a physician working as a pulmonary medicine specialist under GINA guidelines. The diagnosis was made based on the following criteria: asthma medication use, history of various respiratory symptoms, and confirmed variable expiratory airflow limitation [1].
- **Admission to the emergency department**: asthmatic patients who were admitted to the emergency department for health care utilization due to asthma [31].
- **The severity of asthma** was classified based on the GINA 2019 guideline [32].
- **Stages of asthma** were classified as intermittent, mild persistent, moderate persistent, and severe persistent based on the GINA 2019 guideline [32].
- **A physical exercise habit**: a study subject who has experience doing the physical exercise once per day for at least 20–30 min as a continuous activity [33].
- **Elevated blood eosinophil count**: is defined as an absolute eosinophil count greater than 0.04 × 10^3^/µl [34].

#### Data collection and sample processing

The socio-demographic and behavioral characteristics of study participants were collected by using a structured pre-tested questionnaire via face-to-face interview. The clinical data of study participants, such as asthma duration, family history of asthma, history of asthma medication, asthma drug type, chronic disease, severity and stages of asthma, symptoms of asthma, and history of drug intake other than asthma before 3 months, were collected from the patient’s medical record (chart review) by using a checklist. Study participants were sent to the laboratory room, where the blood specimen was drawn and analyzed after trained clinical nurses completed data via face-to-face interview and chart review.

A venous blood specimen (4 milliliters) was collected from asthmatic patients with a syringe method by the trained laboratory technician. Then it is dispensed into the tube containing ethylene-diamine tetra-acetic acid anticoagulant for complete blood count (CBC) and peripheral morphology assessment. Complete blood counts of the study participants were analyzed by using five differential Unicel DxH 800 Coulter cellular analysis systems (Beckman Coulter, Ireland) automated hematological analyzers through Coulter counting, spectrophotometry, and VCS technology.

A peripheral blood morphology examination was performed to confirm flags generated by the automated hematology analyzer and to check whether the automated hematology analyzer provides a consistent result. A thin blood smear was prepared from leftover blood from CBC. Wright stain was used to stain the dried smear. The smear was carefully examined with a 100X oil immersion objective by laboratory technologists. All laboratory tests, such as CBC and peripheral morphology, were performed in the UGCSH and TGSH laboratories. The results of every study participant were recorded in a format designed for laboratory result registration.

#### Data quality assurance and management

The questionnaire was prepared in the English version, translated into the local language (Amharic), and retranslated back to the English version to check the accuracy and consistency. It has been pretested on 5% of asthmatic patients at Felege Hiwot Comprehensive Specialized Hospital. Then a questionnaire was modified based on the result of the pretest. Before the data collection began, training was given for data collectors to reduce technical and observer bias.

Pre-analytical, analytical, and post-analytical phases of quality assurance were maintained in the overall laboratory process to assure the quality of data. Quality control for working equipment and reagents was ensured by using standard controls. Standard operating procedures and manufacturers’ instructions were strictly followed during sample collection and laboratory procedures. The questionnaire was checked for consistency, clarity, and completeness by the principal investigator. The results of every test were properly recorded, reviewed, and transcribed.

#### Data processing and statistical analysis

The data was entered into Epi-data software (Version: 3.0.4), cleaned, and exported into Statistical Package for Social Science version 20 software (IBM Corp., Armonk, NY, USA) for analysis. To summarize the data, descriptive statistics such as frequencies and percentages were used. The Shapiro-Wilk test and histogram were used to check the normal distribution of continuous variables. The data was presented using tables.

Non-parametric (Mann-Whitney test) was used to compare median values of absolute eosinophil count with different background variables. Binary logistic regression, such as bivariable and multivariable logistic regression analysis, was performed. The strength of association between predictors and outcome was determined using the crude odds ratio (COR) and adjusted odds ratio (AOR) with a 95% confidence interval (CI). In the bivariable logistic regression analysis, variables having a p-value of less than 0.25 were fitted into the multivariable logistic regression analysis. Hosmer and Lemeshow’s goodness of fit statistics were used to test the model’s fitness. In all cases, a p-value of less than 0.05 was considered statistically significant.

### Ethical consideration

Ethical clearance was obtained from the ethical review committee of the School of Biomedical and Laboratory Science, College of Medicine and Health Sciences, University of Gondar, on March 3, 2021, with letter reference number SBMLS-2748. The Department of Hematology and Immunohematology provided a support letter, which was forwarded to the directors of UGCSH and TGSH. In addition, a permission letter to conduct research was obtained from the UGCSH and TGSH directors.

Informed written consent was obtained from each study participant after the objective of the study was explained. For illiterate patients, informed consent was obtained after objective of study and detailed information was read by data collectors. Then fingerprint signature was taken from each study illiterate participant. Study participants were identified by using codes rather than individual identifiers. The results and information of the study participants were kept confidential. Study participants who had elevated eosinophil counts were linked to the UGCSH and TGSH chronic outpatient departments for proper management. All methods were performed per the relevant guidelines and regulations (Declaration of Helsinki).

## Result

### Sociodemographic characteristics of the study participants

In this study, we included a total of 291 study participants. Of those, about 164 (56.4%) were females. The age ranges from 18 to 65 years, with the median and interquartile range (IQR) of 50 (38 to 60) years. About 188 (64.6%) of the study participants were over 40 years old. Regarding the residence of study participants, 186 (63.9%) participants were urban residents. The majority of study participants were unable to read and write in their educational status, 126 (43.3%) (Table 1).

**Table 1:** Socio-demographic characteristics of the study participants (n = 291)

Variables	Category	Frequency	Percentage %
Age	≤ 40	103	35.4%
	> 40	188	64.6%
Sex	Male	127	43.6%
	Female	164	56.4%
Residence	Urban	186	63.9%
	Rural	105	36.1%
Marital status	Single	38	13.1%
	Married	192	66.0%
	Divorced	27	9.3%
	Widowed	34	11.7%
Education level	Unable to read and write	126	43.3%
	Primary school	64	22.0%
	Secondary school	49	16.8%
	College and above	52	17.9%
Occupation	House wife	101	34.7%
	Farmer	55	18.9%
	Employer	56	19.2%
	Private worker	57	19.6%
	Non-job, including students	22	7.6%
Family size	≤ 4	139	47.8%
	5–8	132	45.4%
	> 8	20	6.9%

### Behavioral characteristics of study participants and BMI

Of the study participants, 5 (1.7%) were current cigarette smokers; 52 (17.9%) had a habit of doing physical exercise; and 75 (23.4%) had a habit of drinking alcohol. About 64 (22%) had an alcohol-drinking habit. The mean ± standard deviation value of BMI is 2.26 ± 2.97 kg/m^2^. According to their BMI, about 51 (17.5%) of the study participants were overweight.

### Clinical characteristics of study participants

The majority of the study participants had been living with asthma for less than five years, 121 (41.6%). Regarding severity and stages of asthma, about 176 (60.5%) and 186 (63.9%) had moderate severity and moderately persistent stages of asthma, respectively. Among study participants, about 241 (82.8%) had taken asthmatic drugs in the previous three months. In addition, about 86 (29.6%) were admitted to the emergency department (Table 2).

**Table 2:** The clinical characteristics of adult asthmatic patients (n = 291)

Variables	Category	Frequency	Percentage %
Family history of asthma	Yes	88	30.2%
	No	203	69.8%
Duration of asthma	≤ 5	121	41.6%
	6–10	79	27.1%
	> 10	91	71.3%
Severity of asthma	Mild	59	20.3%
	Moderate	176	60.5%
	Severe	56	19.2%
Stage of asthma	Intermittent	36	12.4%
	Mild persistent	19	6.5%
	Moderate persistent	186	63.9%
	Severe persistent	50	17.2%
Asthma medication	Yes	241	82.8%
	No	50	17.2%
History of taking drugs other than asthma	Yes	91	31.3%
	No	200	68.7%
Comorbidities	Yes	87	29.9%
	No	204	70.1%
ED admission	Yes	86	29.6%
	No	205	70.4%
Asthma drugs	ICS	7	3.1%
	ICS-SABA	110	49.1%
	SABA	91	40.6%
	Others	16	7.1%
Systemic corticosteroid use	Yes	17	7.1%
	No	224	92.9%
Symptoms of asthma	Shortness of breath	118	40.5%
	Cough	88	30.2%
	Wheezing	13	4.5%
	≥ 2 symptoms	72	24.7%

**Note: ICS**: Inhaled Corticosteroids, **SABA**: Short-acting beta 2 antagonists, **ICS-SABA**: Inhaled Corticosteroids-Short acting beta 2 antagonists

### Magnitudes of the elevated peripheral eosinophil count and its comparison with background variables

In this study, the magnitude of eosinophilia was 19.6% (95% CI = 14.8–24.1) with a median and IQR of 0.2 (0.07–0.4). The non-parametric Mann-Whitney U test was used to compare median differences in absolute eosinophil counts between groups because the data were not normally distributed. Based on this analysis, only asthma medication, asthma-related ED admission, and intestinal parasitic infection showed a statistically significant median difference (Table 3).

**Table 3:** Comparison of eosinophilia with different background variables (Mann-Whitney test)

Variables	Category	Eosinophil count median (IQR)	p-value
Age	≤ 40	0.2(0.09–0.43	0.211
	> 40	0.18(0.07–0.4)	
Family history of asthma	Yes	0.2(0.065–0.4)	0.134
	No	0.16(0.07–0.4)	
Asthma medication	Yes	0.2(0.075–0.4)	0.011*
	No	0.1(0.07–0.2)	
History of taking drugs other than asthma	Yes	0.12(0.04–0.4)	0.54
	No	0.2(0.1–0.4)	
Comorbidities	Yes	0.19(0.1–0.4)	0.73
	No	0.2(0.06–0.4)	
Admitted to ED	Yes	0.1(0.00–0.19)	0.0001*
	No	0.2(0.1–0.4)	
SC use	Yes	0.1(0.08–0.4)	0.47
	No	0.2(0.035–0.36)	
IP	Yes	0.3(0.1–0.4)	0.045*
	No	0.17(0.07–0.4)	

**Note**: **IQR**: Inter Quartile Range, **ED**: Emergency Department, **SC**: Systemic corticosteroids, **IP**: Intestinal parasite, * shows statistically significant association

### Factors associated with elevated peripheral eosinophil count

A binary logistic regression analysis was performed to check for factors associated with eosinophilia. In bivariable logistic regression analysis, variables like gender, alcohol drinking habit, and asthma-related emergency department admission were shown to have an association with eosinophilia. In addition, the factors with a p-value less than 0.25 were fitted into multivariable logistic regression analysis. Being admitted to the emergency department (AOR = 0.25; 95% CI: 0.09–0.69, p = 0.007) and being female (AOR = 0.49; 95% CI: 0.26–0.9, p = 0.025) were showed a statistically significant association with eosinophilia in a multivariable logistic regression model (Table 4).

**Table 4:** Bivariable and multivariable logistic analysis of factors associated with eosinophilia (n = 291)

Variables	Category	Eosinophilia		COR (95% CI)	P-value	AOR (95% CI)	P-value
		Yes	No				
Age	≤ 40	26(45.6%)	77(32.9%)	1^a^	0.074	1^a^	0.132
	> 40	31(54.4%)	157(67.1%)	0.58(0.32–1.05)		0.62(0.33–1.16)	
Gender	Male	33(57.9%)	94(40.2%)	1^a^	0.017	1^a^	0.025*
	Female	24(42.1%)	140(59.8%)	0.49(0.27–0.88)		0.49(0.26–0.9)	
Alcohol drinking habit	Yes	20(35.1%)	44(18.8%)	2.33(1.24–4.40)	0.009	1.81(0.9–3.54)	0.094
	No	37(64.9%)	190(81.2%)	1^a^		1^a^	
Severity	Mild	9(15.8%)	50(21.4%)	1^a^	0.078	1^a^	0.102
	Moderate	31(54.4%)	145(62%)	1.19(0.53–2.67)		0.81(0.31–2.1)	
	Severe	17(29.8%)	39(16.7%)	2.42(0.97–6.02)		1.79(0.63–5.1)	
Asthmatic drug intake	Yes	51(89.5%)	190(81.2%)	1^a^	0.14	1^a^	0.9
	No	6(10.5%)	44(18.8%)	0.51(0.2–1.26)		1.08(0.32–3.59)	
Emergency department admission	Yes	7(12.3%)	79(33.8%)	0.27(0.12–0.63)	0.002	0.25(0.09–0.69)	0.007*
	No	50(87.7%)	155(66.2%)	1^a^		1^a^	

**AOR**: Adjusted odds ratio **COR**: Crude odds ratio **CI**: Confidence interval **1**^**a**^: Reference group, * statistically significant association

## Discussion

In the present study, eosinophilia was observed in 19.6% (95% CI = 14.8–24.1) of asthmatic patients. This finding was consistent with the study conducted in the USA (18.5%) [35], the United Kingdom (16%) [36], Spain (20.7%) [37], and a cross-sectional survey of the United States general population (18%) [38]. However, the finding was lower than a study done in Japan (34%) [39], a pilot study in the USA (40%) [40], Canada (41%) [41], Brazil (40%) [42], Mexico (37.7%) [43], and the United Kingdom (43%) [44]. The possible reasons for the difference might be due to variation in study participants, socio-demographic characteristics, and sample size. In addition, the recruitment of circulating eosinophils is elevated during allergic conditions, particularly in asthma [45, 46]. It is also known that blood eosinophilia can be a marker of asthma [37].

The comparison of absolute eosinophil count with different background variables was done in the present study. Based on the findings of this study, the absolute eosinophil count was higher among patients who were infected with intestinal parasitic infections than among patients who were not infected with intestinal parasitic infections. This finding was supported by different studies [47, 48]. The possible explanation might be due to the production and activation of eosinophils induced by IL-5, which is secreted by Th2 cells. Thus, eosinophils are involved in host defense, inflammation, and immunomodulation [49]. Eosinophil levels are increased during allergic reactions and helminth infections [50].

In this study, we used binary logistic regression analysis to observe the association between the severity of asthma and eosinophilia. However, severe asthma did not show a statistically significant association with eosinophilia in the present study. This might be due to the fact that the majority of study participants were older (> 40 years old) and females, which are characteristics of adult-onset asthma. This finding was supported by the West Sweden Asthma study (WSAS) and Obstructive Lung Disease in Northern Sweden (OLIN) studies. A 15-year follow-up WSAS study showed that the severity of asthma was associated with blood neutrophilia rather than blood eosinophilia [51, 52]. Amelink et al. also showed that blood eosinophils are not a marker of severity in all asthmatic patients because asthma is a diverse disease with different phenotypes [53].

On the contrary, various studies have found that an elevated eosinophil count is associated with severe asthma [9, 35–37, 54, 55]. This is due to the fact that inflammation is firmly related to the existence of elevated eosinophils in the airway as well as the expression of the Th2 cytokine during allergic asthma. In particular, IL-5 plays a major role in the recruitment, survival, and maturation of eosinophils [11, 56]. Also, it should be known that asthma is a heterogeneous disease with different phenotypes.

Females were less likely to develop eosinophilia than males (AOR = 0.48; 95% CI: 0.26–0.9). This might be because women had a higher incidence of adult-onset asthma when compared to males [57–59]. It is known that in adults, asthma is more often non-allergic than allergic [23, 60]. Thus, T2-low asthma is characterized by neutrophilic airway inflammation through the involvement of non-type 2 cytokines such as IL-8 and IL7 rather than eosinophilic inflammation [29, 61].

Study participants who were admitted to the ED (AOR = 0.25; 95% CI: 0.09–0.69, p = 0.007) were less likely to develop eosinophilia than patients who were not admitted to the ED. The possible explanation might be that the patients attending the ED might be treated with a high dose of inhaled corticosteroids to reduce exacerbation according to GINA guidelines [1, 11, 62]. Eosinophil counts are known to be reduced by ICS’s anti-inflammatory effect [63–65]. ICS inhibits the transcription of protein-coding genes (such as IL-4, IL-5, IL-13, and TNF-α), as well as chemokines and adhesion molecules, reducing inflammatory cell recruitment and survival (like eosinophils, T-lymphocytes, and mast cells) [65]. Moreover, low cytokine production, particularly IL5, reduces the recruitment of eosinophils [66].

This study was limited to showing cause and effect relationships due to the cross-sectional nature of the study. We did not assess the association between the time of oral corticosteroid use and the drug dose with eosinophil level. Another limitation was that the asthma control status of patients was not studied.

In conclusion, the elevated blood eosinophil count was not associated with the severity of asthma among adult asthmatic patients. Lack of eosinophilia can be related to the low-T2 asthma phenotype. Elevated blood eosinophil counts were negatively associated with female sex and ED visits. However, the eosinophil values were higher in asthmatic patients infected with intestinal parasitic infection when compared to their counterparts. Therefore, different biomarkers will be considered in addition to eosinophil count for appropriate diagnosis and therapeutics among adult asthma patients.

## Data Availability

All the data on which the conclusions of this manuscript were drawn are available from the corresponding author. So that anyone who needs the data can get it upon reasonable request.

## List of Abbreviations

ATS: American Thoracic Society
AOR: Adjusted Odds Ratio
BMI: Body Mass Index
CBC: Complete Blood Count
CI: Confidence Interval
COR: Crude Odds Ratio
ED: Emergency Department
ERS: European Respiratory Society
ICS: Inhaled Corticosteroids
IL: Interleukin
IP: Intestinal Parasitic Infection
GINA: Global Initiative for Asthma
OLIN: Obstructive Lung Disease In Northern Sweden
RBC: Red Blood Cell
SABA: Short-acting beta 2 antagonists
SC: Systemic Corticosteroids
SOP: Standard Operating Procedure
SPSS: Statistical Package for Social Science
TGSH: Tibebe-Ghion Specialized Hospital
Th2: T Helper 2
UGCSH: University of Gondar Comprehensive Specialized hospital
WSAS: West Sweden Asthma Study
